# Complete Genome Sequence of the Ice-Nucleation-Active Pseudomonas syringae pv. pisi Isolate MUP32, Isolated from Frost-Damaged Pea (Pisum sativum subsp. *arvense* cv. Dundale) in New South Wales

**DOI:** 10.1128/mra.01276-22

**Published:** 2023-02-13

**Authors:** Hussain Alattas, Julie Ardley, Rebecca Swift, Pragya Kant, Sarah Jackson, Ben Biddulph, Amanuel Bekuma, Jaco Zandberg, Sam Abraham, Ravi Tiwari, Wayne Reeve

**Affiliations:** a Centre for Crop and Food Innovation, Food Futures Institute, Murdoch University, Murdoch, Western Australia, Australia; b School of Molecular and Life Sciences, Faculty of Science and Engineering, Curtin University, Perth, Western Australia, Australia; c Agriculture Victoria, AgriBio, Bundoora, Victoria, Australia; d Department of Primary Industries and Regional Development, Western Australia, Perth, Western Australia, Australia; e Antimicrobial Resistance and Infectious Diseases Laboratory, Harry Butler Institute, Murdoch University, Murdoch, Western Australia, Australia; University of Arizona

## Abstract

The genome of Pseudomonas syringae MUP32, which was isolated from frost-damaged pea in New South Wales, Australia, is tripartite and contains a circular chromosome (6,032,644 bp) and two plasmids (61,675 and 54,993 bp). IMG/M genome annotation identified 5,370 protein-coding genes, one of which encoded an ice-nucleation protein with 19 repetitive PF00818 domains.

## ANNOUNCEMENT

The Pseudomonas syringae species complex (1, 2) is divided into 13 distinct phylogroups, with many strains containing ice-nucleation proteins that contribute to frost damage in crops (3, 4). As part of an ongoing study, we have been identifying bacteria that dominate frost-damaged crops across Australia. Here, we report the complete genome sequence of a P. syringae isolate designated MUP32 (= WAC15092). This strain was isolated (5) from mid-April-sown pea (Pisum sativum subsp. *arvense* cv. Dundale) that had been frost damaged at the reproductive stage and was collected from a frost trial site in Young, New South Wales, Australia, in 1991. Bacteria were isolated and stored in freeze-dried ampoules at the Plant Pathology Laboratory, Agriculture Victoria (Horsham, Australia).

A pure culture of this strain was grown in lysogeny broth (LB) and cryopreserved in 15% glycerol at −80°C. High-molecular-weight genomic DNA was isolated from a logarithmic-phase culture as described previously (6), and the same DNA preparation was sequenced using both Illumina and Oxford Nanopore Technologies (ONT) platforms. ONT libraries were prepared according to the ONT 1D ligation library preparation protocol (SQK-LSK109) and sequenced with a FLO-MIN-106D flow cell (R9.4.1) on a MinION platform. Guppy v3.2.6 was used for base calling with a read-pass-filter quality score cutoff value of 7. Sequencing generated 33,004 reads for a total of 537,421,003 bp (120× coverage), with a read *N*_50_ value of 16,284 bp.

An Illumina library was prepared using the NuGEN Celero DNA-sequencing library preparation kit, following the manufacturer’s protocol. The library was sequenced on an Illumina NextSeq platform using a midoutput 2 × 150-bp kit. This workflow generated a total of 1,262,332 paired-end reads and 184,599,527 bp of sequence (~30× coverage).

**FIG 1:**
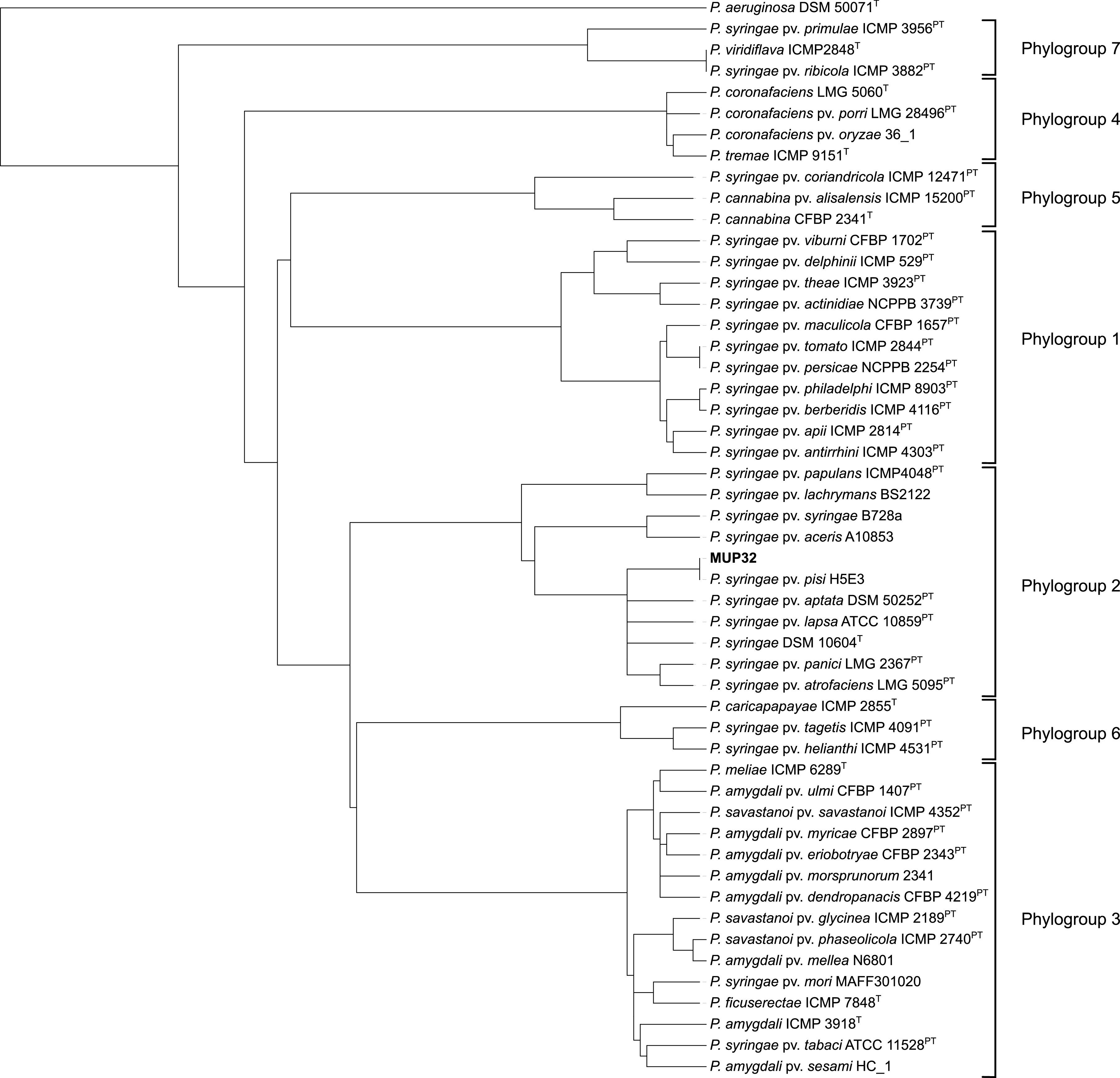
DendroUPGMA-generated tree displaying the relatedness of Pseudomonas genomes based on ANIb values. The ANIb values were generated using FastANI (11) and imported into DendroUPGMA, and the tree was constructed using a similarity matrix within the algorithm (12). The DendroUPGMA-generated tree was exported into Interactive Tree Of Life (iTOL) (13) for visualization. The superscript T indicates type strains, and the superscript PT indicates pathotype and type strains. Pseudomonas aeruginosa DSM 50071^T^ was used as an outgroup.

ONT long reads were assembled using the Flye v2.9 assembler (7) with default parameters, with 10 iterations. Short reads were mapped to the long-read assembly using minimap2 (8) to correct ONT-generated sequencing errors. The final assembly resulted in a single circular chromosome (6,032,644 bp, with a GC content of 58.9%) and two plasmids (61,675 bp, with a GC content of 56.4%, and 54,993 bp, with a GC content of 55.6%). The resulting chromosome was manually oriented using Geneious Prime (9) to position the *dnaA* gene at the first position in the sequence. A quantitative assessment of the genome assembly was performed using BUSCO v5.3.2 (10), which provided a completeness score of 99.6%. A comparison of average nucleotide identity using BLAST (ANIb) (11–13) values for Pseudomonas genomes revealed that MUP32 is a P. syringae (97.8% identity of the genome to the P. syringae type strain DSM 10604 (GenBank accession number NZ_JALK00000000.1)). MUP32 has 99.97% identity of the genome to P. syringae pv. pisi H5E3 and belongs in phylogroup 2 (Fig. 1).

Gene calling and annotation of the generated chromosomal sequence were performed using the IMG/M annotation pipeline (IMGAP) v5.1.8 (14) and the NCBI Prokaryotic Genome Annotation Pipeline (PGAP) (15). A genome annotation comparison is summarized in Table 1.

**TABLE 1:** General features of the genome using different annotation pipelines

Feature	Data from:	
	GenBank annotation	IMG/M annotation
Total no. of genes	5,386	5,534
No. of protein-coding genes	5,166	5,370
No. of rRNA operons	5	5
5S	6	6
16S	5	5
23S	5	5
No. of tRNAs	64	67
No. of other RNA genes	4	4
Locus tag prefix	OQB64_	Ga0569612_01_

Of particular interest was the identification of a gene (locus tag Ga0569612_01_1850483_1854499) encoding an ice-nucleating protein that contained 19 repeats of the ice-nucleation domain PF00818 (16).

### Data availability.

The whole-genome sequence for P. syringae MUP32 has been deposited in the publicly facing Genomes OnLine Database (GOLD) v8 (17) (project identification number Gp0618193), GenBank database (accession numbers CP110809, CP110810, and CP110811 for the chromosome, plasmid pMUP32a, and plasmid pMUP32b, respectively), and IMG/M database (genome identification number 2974445944). The raw sequencing reads have been deposited in GenBank under the accession numbers SRR22226934 and SRR22252530 for long reads and short reads, respectively.
